# Regulation of Interferon Gamma Signaling by Suppressors of Cytokine Signaling and Regulatory T Cells

**DOI:** 10.3389/fimmu.2013.00469

**Published:** 2013-12-18

**Authors:** Joseph Larkin, Chulbul M. Ahmed, Tenisha D. Wilson, Howard M. Johnson

**Affiliations:** ^1^Department of Microbiology and Cell Science, University of Florida, Gainesville, FL, USA

**Keywords:** auto-immunity, T cells, tolerance/suppression/anergy, inflammation, lupus

## Abstract

Regulatory T cells (Tregs) play an indispensable role in the prevention of autoimmune disease, as interferon gamma (IFNγ) mediated, lethal auto-immunity occurs (in both mice and humans) in their absence. In addition, Tregs have been implicated in preventing the onset of autoimmune and auto-inflammatory conditions associated with aberrant IFNγ signaling such as type 1 diabetes, lupus, and lipopolysaccharide (LPS) mediated endotoxemia. Notably, suppressor of cytokine signaling-1 deficient (SOCS1^−/−^) mice also succumb to a lethal auto-inflammatory disease, dominated by excessive IFNγ signaling and bearing similar disease course kinetics to Treg deficient mice. Moreover SOCS1 deficiency has been implicated in lupus progression, and increased susceptibility to LPS mediated endotoxemia. Although it has been established that Tregs and SOCS1 play a critical role in the regulation of IFNγ signaling, and the prevention of lethal auto-inflammatory disease, the role of Treg/SOCS1 cross-talk in the regulation of IFNγ signaling has been essentially unexplored. This is especially pertinent as recent publications have implicated a role of SOCS1 in the stability of peripheral Tregs. This review will examine the emerging research findings implicating a critical role of the intersection of the SOCS1 and Treg regulatory pathways in the control of IFN gamma signaling and immune system function.

## Introduction

The primary role of the immune system is to eliminate pathogenic, infectious agents and altered self (cancers) (1–3). Interferon gamma (IFNγ) plays a critical role in the capacity of the immune system to recognize and eliminate pathogenic agents through increased presentation of antigens by antigen presenting cells, induction of an anti-viral state in infected cells, and mediation of anti-microbial effector functions (4). In addition, IFNγ serves to mediate the destruction of cancerous cells by inducing an anti-proliferative state, enhancing NK cell activity, and mediating antibody production (4–6). Conversely, aberrant immune system functions can result in the destruction of self-tissues (auto-immunity) and dysbiosis of natural gut flora communities (1, 7). It is therefore essential that IFNγ, a central effector of the immune system, be carefully regulated. Foxp3^+^ regulatory T cells (Tregs), and the intracellular protein suppressor of cytokine signaling-1 (SOCS1), are two critical regulators of IFNγ signaling. In this review we will: (1) define the role of these two mechanisms in the regulation of IFNγ signaling, (2) examine evidence suggesting cross-talk between these two pathways, and (3) explore emerging research whereby IFNγ signaling is regulated through the use of SOCS1 mimetic peptides.

## IFN Gamma Signaling

The sole type II interferon, IFNγ is a multipotent cytokine secreted by activated T cells and natural killer cells and is responsible for the modulation of many facets of immune response. IFNγ exerts its effects through interactions with the IFNγ receptor complex (IFNGR) on the surface of target cells. The IFNGR is a heterodimeric complex that consists of an alpha subunit IFNGR1 (90 kDa) and a beta subunit IFNGR2 (60 kDa) (8, 9). IFNGR1 binds to IFNγ with high affinity whereas IFNGR2, although contributing to ligand binding and required for signaling, binds to IFNγ with a significantly lower affinity (9, 10). The binding involves crosslinking two molecules each of IFNGR1 and IFNGR2. Subsequent to ligand binding, tyrosine kinases JAK1 (constitutively associated with IFNGR1) and JAK2 (constitutively associated with IFNGR2) become phosphorylated and subsequently activate STAT1 through phosphorylation (9, 11, 12). It is then thought that phosphorylated, activated STAT1 translocates to the nucleus, where it mediates the transcription of IFNγ associated proteins. The immediate effects of IFNγ signaling occur rapidly as JAK1, JAK2, IFNGR1, and Stat1 are phosphorylated within 1 min of IFNγ treatment (4).

## IFNγ and Disease

Although IFNγ signaling is critical for the generation of effector functions involved in the elimination of cancers and pathogenic microorganisms, the signaling must be carefully regulated to prevent excessive inflammation. The potential deleterious effects of IFNγ signaling are also underscored by the fact that administration of IFNγ to neonatal mice results in a lethal auto-inflammatory disease (13, 14). Significantly, excessive IFNγ signaling has been associated with several autoimmune diseases including systemic lupus erythematosus (SLE) in both patients and rodent models of disease (15–18). SLE is a debilitating autoimmune disease with numerous medical manifestations including leucopenia, skin rashes, and damage to heart and kidneys. Moreover, ongoing research strategies for treatment of autoimmune diseases such a lupus include the use of antibodies targeting cytokines involved in disease progression, such as IFNγ (15).

In addition to tolerance to self-tissues, the immune system must also maintain a balance between the elimination of infectious microorganisms and the tolerance of mutualistic microorganisms, which both regulate mammalian metabolic events and immune system development (19). Lipopolysaccharide (LPS) endotoxin, by Gram negative bacteria, and Staphylococcal enterotoxins (SE), by Gram positive bacteria, pose significant risks to human health by their capacity to dysregulate immune tolerance mechanisms and generate a lethal inflammatory environment, with excess production of IFNγ (20). This is especially true in the case of combination challenges (infections are commonly polymicrobial), as it has been established that the combination challenges of Staphylococcal enterotoxin A (SEA) and LPS mediate more severe inflammatory environments in intact mice (20). SEA, which is a member of the superantigen toxins produced by *Staphylococcus aureus*, activates T lymphocytes possessing T cell receptors (TCR) of varying V-beta specificities, and is capable of mediating toxic shock syndrome. SEA crosslinks the antigen presenting cell MHC molecule, with the TCR V-beta chain, resulting in the production of abundant pro-inflammatory cytokines such as tumor necrosis factor alpha (TNFα), IFNγ, interleukin 1 (IL1), and IL6 (20). A hallmark of the adaptive immune system is antigenic specificity (21). Whereas a conventional T cell antigen mediates the activation of T lymphocytes on the order of one T cell clone/10,000 lymphocytes, as many as one in five T lymphocytes can be activated by the superantigen (22). Moreover the excessive activation of T lymphocyte clones occurs at a fraction of the concentration required to mediate conventional T lymphocyte activation. Notably, staphylococcal superantigens account for nearly 45% of all cases of food poisoning (23). IFNγ and IL6 produced in response to microbial insult contribute to fever, nausea, vomiting, and diarrhea associated with the food poisoning. Given the potential for IFNγ signaling to result in auto-immunity or excessive responses directed toward microorganisms, the immune system has developed regulatory mechanisms to limit IFNγ signaling, which are potential targets of modulation in excessive inflammatory environments.

## Regulatory T Cells and Auto-Immunity

Despite the fact that T cells bearing specificity to self-peptides exist in nearly all individuals, autoimmune diseases are in limited numbers when compared to other sources of human disease (24, 25), thus underscoring the presence of tolerance mechanisms. Beyond central tolerance, one mechanism of active immune tolerance is Tregs. Although several distinct T cell populations possessing regulatory function have been described, CD4^+^ Tregs bearing the Foxp3^+^ transcription factor and constitutive expression of CD25 represent one of the lineages most characterized to date (26–28). However, it is important to note that Foxp3 alone is not sufficient to recapitulate the entire Treg signature (29), suggesting that additional Treg modulating genes likely remain to be characterized. Tregs have the capacity to suppress immune responses mediated by numerous cell types including macrophages, CD4^+^ T cells, and CD8^+^ T cells (30–34). The importance of functional Tregs in the prevention of auto-immunity is underscored by the fact that the adoptive transfer of Tregs into type 1 diabetes (35), experimental allergic encephalomyelitis (36), inflammatory bowel disease (37), and arthritis (38) rodent models resulted in the prevention/cure of disease.

Regulatory T cells, under the pseudonym suppressor T cells, have origins within the pioneering studies of Gershon followed by a rebirth powered largely by the Shevach and Sakaguchi laboratories (39). Tregs, along with other T lymphocytes, originate from the thymus as illustrated in seminal studies showing that day 3 thymectomy of neonatal mice resulted in the development of organ specific autoimmune diseases, which could be prevented by the administration of CD4^+^CD25^+^ T lymphocytes from healthy mice. Naturally occurring, thymic derived Foxp3^+^ Tregs constitute approximately 3–5% of the CD4^+^ CD8^−^ thymocyte population (40) and are generally present within the medullary region of the thymus, the location of the most mature thymocytes (41). Further advances in the characterization of Tregs were made after the discovery that the lethal pathology observed in *Scurfy* mice was due to defective Foxp3. *Scurfy* mice, which lack CD4^+^ CD25^+^ Foxp3^+^ Treg, die of a perinatal auto-inflammatory disease within 21 days after birth (39). Moreover, *Scurfy* mice die of massive infiltrations of activated leukocytes in multiple organs with autoimmune disease manifestations including lymphadenopathy, splenomegaly, anemia, and wasting (39). Notably, depletion of Th1 responses through the breeding of *Scurfy* mice to IFNGR1 or *Tbet* deficient backgrounds, results in prolonged survival of *Scurfy* mice (42). These results underscore the role that Tregs serve in critically regulating Th1 responses.

To date, there are two characterized populations of Foxp3^+^ Tregs: naturally occurring Tregs that develop in the thymus (nTregs); and induced Tregs (iTregs) which are generated within peripheral organs from naïve CD4^+^ CD25^−^Foxp3^−^ T lymphocytes (39, 43–45). Both nTregs and iTregs are thought to play a significant role in the regulation of immune responses, with nTregs focused on immunity directed against self-peptides and iTregs limiting immune pathology in response to commensal bacteria. Markers used to distinguish iTregs from nTregs include neuropilin-1 (46) and the transcription factor Helios (47). Generation of peripheral Tregs can be mediated through the expansion of nTregs in the presence of endogenous peptides specific to Treg TCR, administration of low levels of cognate antigen, or transforming growth factor β (TGFβ) administration (48–51).

The survival and function of Tregs is critically dependent upon cytokines, such as Interleukin 2 (IL2) which is required for the survival of nTregs (52), and enhanced peripheral generation of iTregs (53, 54). In contrast, IL6 inhibits the generation of iTregs while favoring the generation of IL-17 secreting cells (55). Although Tregs can inhibit the production of IFNγ, Th1 associated cytokines IL27 and IFNγ have been shown to increase TGF-β induced Foxp3 expression (56, 57). However, other studies have shown that a Th1 response inhibits the generation of peripheral Tregs (58). It is possible that the ability of IFNγ to up-regulate the IL2 receptor (CD25) (59) on lymphocytes may contribute to the increased FoxP3 expression observed, although further studies are necessary. It is thus clear that the differentiation, survival, and regulation of Tregs is dependent upon complex interactions with cytokines. Conversely, it is also clear that the regulation of inflammatory cytokines, such as IFNγ, is also critically dependent on Tregs.

## Regulation of IFNγ Signaling through SOCS Proteins

In addition to regulation of IFNγ signaling by Tregs, it has become evident that the SOCS family of intracellular proteins also plays a pivotal role in such signaling (60). The SOCS protein family, identified in 1998, currently possesses eight family [SOCS1 to SOCS7 and cytokine-inducible SH2 protein (CIS)] members, which negatively regulate cellular responses to cytokines in a feedback inhibition fashion (61). SOCS proteins are induced by several cytokines and act to inhibit the signaling of the cytokine that mediated their generation (62). SOCS proteins act through at least two mechanisms: (1) SOCS1 possesses a kinase inhibitory region (KIR) that binds to JAKs, thus inhibiting further cytokine signaling and (2) SOCS1 contains a region known as the SOCS box, which targets bound proteins to the proteasome for degradation (60). Whereas all of the SOCS proteins possess a large central SH2 domain and a C-terminal SOCS box, the N-terminal (12-amino acid long, adjacent to the SH2 domain) KIR region has only been identified in SOCS1 and SOCS3. While SOCS1 was initially identified as a regulator of IL6 signaling (63), it later became identified as a regulator of IFNγ. Mice lacking SOCS1 (SOCS1^−/−^) die within 21 days after birth, and are characterized by excessive IFNγ signaling and massive inflammatory infiltration of auto-immune leukocytes into several organs including lungs, pancreas, liver, and heart (63). Neonatal lethality in SOCS1^−/−^ mice is believed to be due to excessive IFNγ signaling, as the treatment of SOCS1^−/−^ mice with IFNγ neutralizing antibodies or breeding to an IFN gamma deficient background rescues mice from neonatal lethality (64). SOCS1 deficient mice, made transgenic to express SOCS1 protein possessing the KIR region, but lacking the SOCS box, could survive peri-lethality with approximately 20% surviving long-term (65). Functionally, the regulation of IFNγ signaling by SOCS1 is mediated by inhibition of JAK2 activity, but potentially also through the regulation of tyrosine kinases TYK2 and JAK1.

## SOCS1 and Auto-Immunity

In addition to the lethal, auto-inflammatory disease that occurs within SOCS1-KO mice, deficiencies in SOCS1 are also specifically linked to the onset and progression of SLE. SOCS1^−/−^ mice, with forced expression of SOCS1 in B and T lymphocytes, and SOCS1^+/−^ mice survive perinatal lethality but develop a lupus-like disease (66, 67). The F1 progeny of New Zealand Black and New Zealand White (NZB/W) mice are a well-established model for spontaneous SLE (68). Notably, splenocytes present in diseased NZB/W mice have a reduced capacity to up-regulate SOCS1 in response to IFNγ (16). Moreover, the administration of a tolerogenic peptide, hCDR1, prevented lupus onset in NZB/W mice and enhanced production of SOCS1. In addition, the enhanced SOCS1 expression was correlated to reductions in IFNγ production and STAT 1 activation in NZB/W mice (16). Although there is good evidence in rodent studies denoting a role for SOCS1 expression in the prevention of murine lupus, more studies are necessary to establish the translational value of this finding.

Moreover it has been shown that SOCS1 signaling plays an important role in moderating signaling directed toward microbial antigens, or microbial lymphocyte mitogens, as deficiencies in SOCS1 result in hyper-responsiveness to LPS (69). Although mice are more resistant to the individual effects of SEA or LPS than humans, immunological tolerance mechanisms regulating lethal inflammation in mice are overcome when co-challenged with SEA and LPS (20). Significantly, single administration of LPS to SOCS1 deficient mice resulted in lethal inflammation (69, 70), underscoring the critical role of SOCS1 in the maintenance of tolerance and prevention of lethal inflammation.

## Treg/SOCS1 Cross-Talk Regulation of IFNγ Signaling

**Figure 1:**
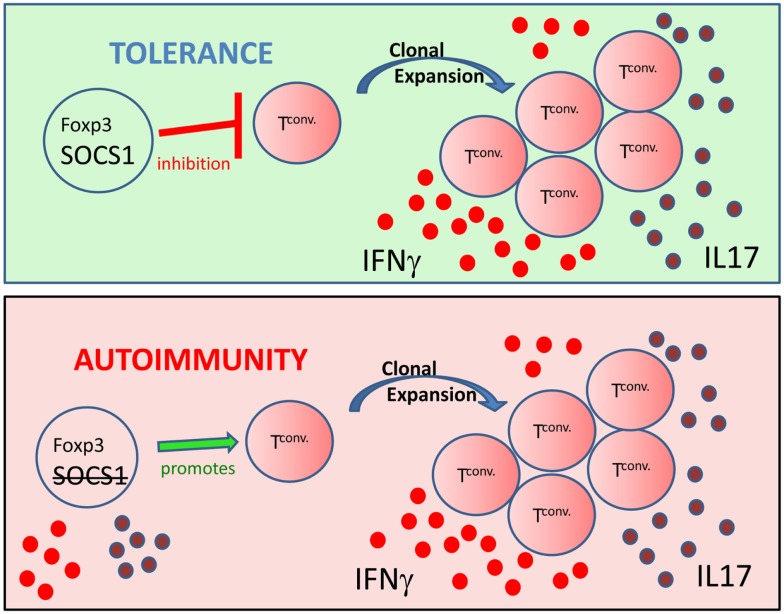
**From inhibitor to contributor: figure representing the critical role of SOCS1 in the stability and function of Foxp3^+^ Tregs**. (Top) Tregs possessing Foxp3 and SOCS1 inhibit clonal expansion and the production of inflammatory effector cytokines such as IFNγ (red circles) and IL-17 (purple circles) by conventional T cells (Tconv), thus maintaining immune homeostasis. (Bottom) T cells possessing Foxp3, but lacking SOCS1, produce inflammatory cytokines and promote Tconv generation of an inflammatory environment. Thus exTregs expressing Foxp3, but lacking SOCS1, can promote auto-immunity.

Auto-immunity is due largely to the cumulative effects of auto-antibody production (71), dysregulated pro-inflammatory cytokine signaling (72), and decreased regulatory T cell function (Tregs) (73, 74). It is likely, however, that the cumulative effects promoting auto-immunity are somewhat interdependent as we have previously shown that Tregs can inhibit auto-reactive T cell proliferation (75), auto-antibody production (34), and pro-inflammatory cytokine production (76). In similar fashion, immune system regulatory mechanisms are also interconnected. For example, thymic events not only mediate central tolerance, which limits the export of self-reactive T lymphocytes into the periphery, but also is the location o f nTreg development. Notably, several recent studies have shown that SOCS1 and Foxp3^+^ Treg regulation of inflammation are also interconnected. Similar to *Scurfy* mice, survival of SOCS1^−/−^ mice was greater with the adoptive transfer of wild-type conventional cells combined with Tregs compared to transfers of Tregs alone (77, 78). In addition, *Scurfy* and SOCS1 deficient mice both have significantly expanded populations of peripheral CD44^hi^CD4^+^ T lymphocytes (42, 64). The presence of CD44^hi^ cells is indicative of homeostatic proliferation. Moreover, recent microRNA (miR) studies link Tregs and SOCS1. MicroRNA 155 (miR-155) is highly expressed in Tregs, and is regulated by the transcription factor Foxp3 (79). Foxp3 binds to the promoter region of miR-155 host gene *bic*, mediating miR-155 up-regulation, which is then thought to down-regulate SOCS1 expression (80, 81). High levels of Foxp3 therefore drive up miR-155 expression, down-modulate SOCS1, and enhance the thymic development of Tregs (78, 81, 82). Notably, however, miR-155 is not required for peripheral survival of Tregs or suppressor function (83). Although Rag gene deficient mice are free of spontaneous colitis, SOCS1^−/−^Rag2^−/−^ mice develop severe colitis (84). However, the adoptive transfer of IL10-producing-Tregs is sufficient to prevent colitis in SOCS1^−/−^Rag2^−/−^ mice (84), again underscoring the interconnected nature of SOCS1 signaling and regulatory Tregs in the regulation of immune responses. Significantly, onset of lethal auto-inflammatory disease in SOCS1^−/−^ mice is correlated with a decrease in Foxp3^+^ Tregs, thereby suggesting a critical role of SOCS1 in the peripheral stability of Tregs (78). Moreover, in the absence of SOCS1, Foxp3^+^ T cells produce the inflammatory cytokines IL-17 and IFNγ (85). Further, recent studies show that Ubc13, lysine specific ubiquitin-conjugating enzyme, regulates SOCS1 expression and IL10 production in Tregs (86). This finding is significant as Ubc13 mutation does not affect Foxp3 expression in T lymphocytes; however, Ubc13 deficient Foxp3^+^ T lymphocytes acquire IFNγ or IL-17 producing inflammatory phenotypes (86). Inflammation is regulated through two important arms of the immune system; the intracellular proteins SOCS1 (60, 63) and Foxp3^+^ Tregs (52). Significantly, the studies presented herein implicate a role of SOCS1 in the stability of Tregs, suggesting that loss of SOCS1 function also inhibits Treg function (Figure 1).

## SOCS Mimetics as Therapeutics Against Auto-Immunity and Inflammation

Given the critical importance of SOCS1 in moderating IFNγ signaling directly, and in stabilizing the Treg phenotype (78, 86), methods to modulate SOCS1 function would likely have significant therapeutic potential. Two factors limit the direct use of SOCS1 protein as a therapeutic: (1) SOCS1 is extremely difficult to produce in pure form and (2) since it functions intracellularly, there is also the challenge of it being internalized by cells so that interaction with JAKs can occur.

We have addressed these problems with SOCS1 by development of small peptide SOCS1 mimetics. The focus has been on the 12-amino acid KIR of SOCS1 where we initially developed a 12-residue peptide based on a hydropathic complementary algorithm (87). The resultant tyrosine kinase inhibitory peptide (Tkip), WLVFFVIFYFFR, specifically bound to the activation loop sequence, ^1001^LPQDKEYYKVKEP, of JAK2 (88). Tkip blocked autophosphorylation of JAK2 as well as that of JAK2 phosphorylation of receptor subunit IFNGR1 and the corresponding transcription factor STAT1α. Tkip also blocked phosphorylation of the epidermal growth factor tyrosine kinase receptor (EGFR), whose function is also inhibited by SOCS1 (87). Like SOCS1, however, Tkip did not inhibit receptor tyrosine kinase VEGF kinase activity (87). Attachment of a lipophilic palmitate residue to Tkip (lipo-Tkip) allowed cell penetration for study of function. In this regard, cells treated with lipo-Tkip showed inhibition of IFNγ induced anti-viral activity against EMC virus in WEHI-3 cells and down-regulation of IFNγ induced expression of MHC class I on WISH cells (87).

Tkip protected mice against experimental auto-immune encephalomyelitis (EAE), a mouse model of multiple sclerosis, both in terms of induction of acute EAE, relapsing/remitting EAE, and chronic EAE (89). Importantly, it also showed therapeutic efficacy when treatment was initiated after appearance of paralytic symptoms and central nervous system (CNS) pathology (89). These studies involved injection of lipo-Tkip at 63 μg/dose IP every other day and were the first to show that a SOCS1 mimetic had therapeutic potential in treating an auto-immune disorder.

As indicated, Tkip was developed based on the hydropathic complementary algorithm without an initial consideration of SOCS1-KIR. However, of three sites of homology between Tkip and KIR, residues F56 and F59 of KIR are of particular interest as mutational analysis has shown that they are critical for KIR binding to JAK2 (88). We thus synthesized SOCS1-KIR and determined its binding to JAK2 activation loop, JAK2(1001–1013), with phosphorylated and non-phosphorylated tyrosine 1007 (88). SOCS1-KIR bound to phosphorylated activation loop, pJAK2(1001–1013), with higher affinity than to unphosphorylated activation loop. Unlike Tkip, however, SOCS1-KIR did not block JAK2 autophosphorylation, but like Tkip it did block STAT1α phosphorylation. Thus, Tkip and SOCS1-KIR both recognized JAK2 activation loop, but not in the same precise manner.

We synthesized lipo-SOCS1-KIR and determined its therapeutic efficacy in a murine relapsing/remitting model of EAE. Treatment of SJL/J mice with SOCS1-KIR beginning 12 days post-immunization with myelin basic protein (MBP) resulted in minimal symptoms of EAE, while most control treated mice developed paraplegia (90). Th1 and Th17 cells via IFNγ and IL-17, respectively, are thought to play critical roles in the pathogenesis of EAE and multiple sclerosis (91). SOCS1-KIR treatment suppressed interleukin-17A (IL-17A) production by MBP-specific lymphocytes, as well as MBP-induced lymphocyte proliferation. When treated with IL-23, a key cytokine in the terminal differentiation of IL-17-producing cells, MBP-sensitized cells produced IL-17A and IFNγ; SOCS1-KIR was able to inhibit the production of these cytokines (90). SOCS1-KIR also blocked IL-23 and IL-17A activation of STAT3. There is a deficiency of SOCS1 and SOCS3 expression in CD4^+^ T cells that infiltrate the CNS in EAE, reflecting a deficiency in regulation. Consistent with therapeutic efficacy, SOCS1-KIR reversed the cellular infiltration of the CNS that is associated with EAE (90). These results with an auto-immune neurological model suggest the potential of SOCS mimetics as therapeutics for auto-immune and inflammatory disorders.

The immuno-modulatory potential of lipo-SOCS1-KIR was also examined in SOCS1^−/−^ mice, which die within 21 days of birth of an IFNγ mediated, auto-inflammatory disease (78). Significantly, lipo-SOCS1-KIR administration, in combination with CD4 T cell adoptive transfer, was sufficient to extend the survival of SOCS1^−/−^ mice (78). The survival of the SOCS1^−/−^ mice receiving the combined treatment was similar to previous studies utilizing mice where SOCS1 contained a KIR region, but lacked a SOCS box (65). Moreover the SOCS1-KIR/CD4 T cell adoptive transfer treatment, decreased leukocytic infiltrations of organs, decreased inflammation overall, and decreased IFNγ serum levels. In particular, the combined treatment mediated a very noticeable increase of peripheral Foxp3^+^ Tregs, both number and frequency, which correlated with the increased survival of SOCS1^−/−^ mice (78). This study suggests that administration of mimetics of SOCS1 may potentially enhance Treg mediated regulation of the immune response.

There is also evidence that SOCS1-KIR has therapeutic potential in auto-immune/inflammatory skin disorders such as psoriasis (92). First, others have confirmed the mimetic activity of SOCS1-KIR and have made modifications in residues for a SOCS1-KIR variant that possessed specific activity similar to that of SOCS1-KIR (93). IFNγ plays a key role in psoriasis related pathogenesis of the skin via its activation of keratinocytes (94). Overexpression of SOCS1 in keratinocytes has previously been shown to reduce the inflammation and to restore homeostasis to the skin (95). The SOCS1-KIR mimetic variant inhibited IFNγ induced inflammation in human keratinocytes, which was reflected by inhibition of JAK2, IFNGR1, and STAT1α phosphorylation. The inhibition at the level of JAK2 autophosphorylation suggests that the SOCS1-KIR variant (called PS-5) functioned similarly to that of Tkip (92). Consistent with reduced phosphorylation, PS-5 reduced ICAM-1, HLA-DR, CXCL-10, and CCL-2 inflammatory gene expression. These SOCS1 mimetic results suggest the potential of treatment of psoriasis-type disorders with SOCS1 mimetics.

## From SOCS1-KIR to a SOCS1 Antagonist

The fact that KIR of SOCS1, SOCS1-KIR, can bind to a peptide, pJAK2(1001–1013), that corresponds to the activation loop of phosphorylated JAK2, pJAK2, raised the possibility that pJAK2(1001–1013) could function as an antagonist of SOCS1 (88). We have thus demonstrated under four different types of experiments that pJAK2(1001–1013) possessed SOCS1 antagonist properties. First, pJAK2(1001–1013) enhanced suboptimal IFNγ activity. Second, prostate cancer cells transfected for constitutive production of SOCS1 protein had reduced activation of STAT3 by IL6 treatment. pJAK2(1001–1013) reversed the SOCS1 effect. Third, pJAK2(1001–1013) enhanced IFNγ activation of the luciferase reporter gene via the GAS promoter. Fourth, pJAK2(1001–1013) enhanced antigen-specific splenocyte proliferation. Treatment of cells with IFNγ resulted in activation of the *SOCS-1* gene in approximately 90 min and it has been proposed that this is associated with the physiological attenuation of the IFNγ response by SOCS1 (64). Consistent with this, it has been reported that siRNA inhibition of SOCS1 expression in bone marrow dendritic cells resulted in enhanced cytotoxic T lymphocyte (CTL) activity and IFNγ production by ELISPOT, culminating in enhancement of antitumor immunity (96).

Recently, the SOCS1 antagonist was shown to enhance antigen-presentation and CTL activity of human dendritic cells (97). Specifically, pJAK2(1001–1013) up-regulated the expression of the maturation marker (CD83) and co-stimulatory molecule (CD86) of monocyte-derived mature dendritic cells (mDCs), potentiated the capacity of mDCs to induce T cell proliferation, stimulated the secretion of pro-inflammatory cytokines, and enhanced the cytotoxicity of tumor cell antigen-specific CTLs activated by human gastric cancer cell total RNA-electroporated mDCs. At the level of transcription, pJAK2(1001–1013) enhanced STAT1 activation.

**Figure 2:**
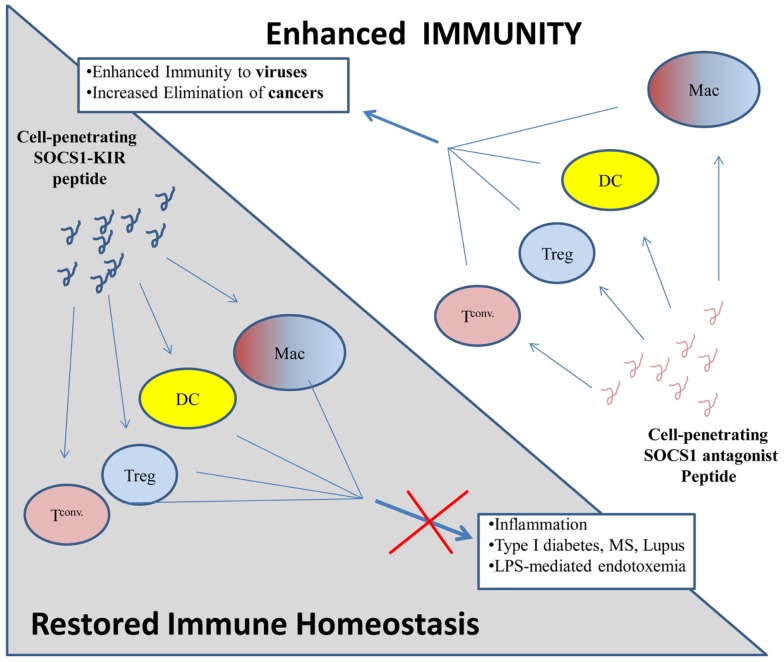
**Proposed model of immune regulation by SOCS1 mimetic peptides and antagonists**. (Top) Cell penetrating SOCS1 antagonist peptides (pink loop) are internalized by various immune cells: macrophages (Mac), dendritic cells (DCs), regulatory T cells (Treg), and conventional T cell (T conv) where they *antagonize* the immuno-suppressive effects of native SOCS1. Inflammatory processes are enhanced, thus facilitating the elimination of cancers and viral infections. (Bottom) Cell penetrating SOCS1-KIR, which mimics the function of native SOCS1 (blue loop), is internalized by various immune cells (as above) where it *enhances* the immuno-suppressive effects of native SOCS1. Inflammatory processes are inhibited by cell penetrating SOCS1-KIR, thus inhibiting diseases which require dysregulated inflammation.

Thus, just as the SOCS mimetics can inhibit inflammatory cytokine activity in auto-immunity, the SOCS1 antagonist can enhance immune activity for treatment of infections and cancers (Figure 2).

## Conclusion

The immune regulatory mechanisms of Tregs and SOCS1 play a critical role in maintaining the balance between the effective clearances of pathogens and maintaining tolerance to self-tissues and mutualistic microorganisms. Notably, recent studies implicate a significant role of SOCS1 in the peripheral survival and phenotypic stability of Tregs, suggesting that defects in the SOCS1 arm of immune homeostasis will also cause defects in the Treg arm of homeostasis. Moreover, the use of peptides which mimic SOCS1 signaling may have therapeutic efficacy in the treatment of auto-immunity by restoring the SOCS1 and Treg immuno-modulatory pathways.

## Conflict of Interest Statement

The authors declare that the research was conducted in the absence of any commercial or financial relationships that could be construed as a potential conflict of interest.
