
JOURNAL INFORMATION
==============================
NLM Title Abbreviation: Front Immunol
Journal ID: Front Immunol
NLM Journal ID: 101560960
Journal ID: Front. Immunol.

Frontiers in Immunology

EISSN: 1664-3224
Publisher: Frontiers Media SA

ARTICLE INFORMATION
==============================
PMCID: PMC3940931
DOI: 10.3389/fimmu.2014.00088
Article version: 1
Subjects: Immunology, General Commentary

Erratum: Regulation of Interferon Gamma Signaling by Suppressors of Cytokine Signaling and Regulatory T Cells

Larkin Joseph III 1 *
Ahmed Chulbul M. 1
Wilson Tenisha D. 1
Johnson Howard M. 1
1 Department of Microbiology and Cell Science, University of Florida , Gainesville, FL , USA
Edited and reviewed by: Lee Mark Wetzler, Boston University School of Medicine, USA

*Correspondence: jlarkin3@ufl.eduThis article was submitted to Immunotherapies and Vaccines, a section of the journal Frontiers in Immunology.

Electronic publication date: 2014 Mar 4
Publication date: 2014
Volume: 5
Electronic Location ID: 88
Received 2014 Feb 14; Accepted 2014 Feb 19
Copyright: Copyright © 2014 Larkin III, Ahmed, Wilson and Johnson.
Copyright year: 2014
License: This is an open-access article distributed under the terms of the Creative Commons Attribution License (CC BY). The use, distribution or reproduction in other forums is permitted, provided the original author(s) or licensor are credited and that the original publication in this journal is cited, in accordance with accepted academic practice. No use, distribution or reproduction is permitted which does not comply with these terms.
License URL: https://creativecommons.org/licenses/by/3.0/

Erratum for: Regulation of Interferon Gamma Signaling by Suppressors of Cytokine Signaling and Regulatory T Cells 4 18 12 2013 469 Frontiers in Immunology PMC3866655 24391643
Figure count: 0
Table count: 0
Equation count: 0
Reference count: 0
Page count: 1

==============================
The author’s own work is supported by a grant from the Lupus Research Institute (Joseph Larkin III), NIH grants R01NS051245 and R01AI056152 (to Howard M. Johnson), a diversity supplement awarded to parent grant R01AI056152, a BD Biosciences Research Grant, the NIH/NCATS Clinical and Translational Science Awards to the University of Florida TL1 TR000066 and UL1 TR000064, and the University of Florida.

